# Anti-inflammatory effect of Chang-An-Shuan on TNBS-induced experimental colitis in rats

**DOI:** 10.1186/s12906-017-1794-0

**Published:** 2017-06-15

**Authors:** Hong Mi, Feng-bin Liu, Hai-wen Li, Jiang-tao Hou, Pei-wu Li

**Affiliations:** 1 Department of Gastroenterology, the First Affiliated Hospital of Guangzhou University of Chinese Medicine, Guangzhou, 510405 China; 2 First School of Clinic Medicine, Guangzhou University of Chinese Medicine, Guangzhou, 510405 China

**Keywords:** Chang-An-Shuan, Ulcerative colitis, Interleukin-1β, Tumor necrosis factor-α, Nuclear factor-κB

## Abstract

**Background:**

Inflammatory bowel disease (IBD), denominated by Crohn’s disease and ulcerative colitis, is often associated with abdominal pain, diarrhea and bloody stool. The standard protocols for treating colitis conditions are not satisfactory; thus, complementary and alternative medicines have been increasingly accepted by IBD sufferers worldwide. In this study, we aimed to elucidate the anti-inflammatory effect of Chang-An-Shuan (CAS), a 6-herb Chinese medicinal formula, on 2, 4, 6-trinitrobenzenesulfonic acid (TNBS)-induced colitis in rats and the underlying mechanisms.

**Methods:**

Sprague-Dawley rats were administered with rectal gavage of 2.5% TNBS in 50% ethanol for the induction of experimental colitis which is considered as a model for Crohn’s disease. Upon the TNBS induction, rats were given CAS at 0.5 g/kg/day or 5 g/kg/day for 10 days. The application of salicylazosulfapyridine (0.5 g/kg/day) was served as a positive reference drug for the colitis condition. The efficacy and mechanistic action of CAS were evaluated by means of histopathological and biochemical approaches such as histological staining, real-time polymerase chain reaction, Western blotting analysis and enzyme-linked immunosorbent assay.

**Results:**

Oral administration of CAS at 5 g/kg/day, but not 0.5 g/kg/day, significantly ameliorated the severity of TNBS-induced colitis as evidenced by the reduced loss of body weight, alleviated diarrhea and decreased bloody stool. While lowering the disease activity index, the administration of CAS lessened mucosal lesions thus mucosal integrity of the colitis rats was notably improved. Further, the CAS treatment also significantly suppressed the mRNA and protein levels of pro-inflammatory cytokines, namely interleukin-1β and tumor necrosis factor-α while enhancing the level of anti-inflammatory cytokine IL-10 in the TNBS-treated rats. Importantly, the ameliorative effect of CAS was related to an inhibition of the nuclear factor-κB (NF-κB) signaling pathway by downregulating the expression levels of NF-κBp-65, p-38 and p-AKT.

**Conclusions:**

We suggest that CAS is a potential alternative remedial approach for treating IBD conditions, and the anti-inflammatory effect of CAS is associated with the down-regulation of the NF-κB signaling pathway and the balanced production of pro- and anti-inflammatory cytokines.

## Background

Inflammatory bowel disease (IBD), denominated by Crohn’s disease (CD) and ulcerative colitis (UC), is an immune-mediated chronic intestinal disorder characterized by rectal bleeding, mucous stool and diarrhea, in which epithelial barrier disruption and mucosal ulceration are associated [1]. The mainstay therapies for colitis include anti-inflammatory drugs, 5-aminosalicylic acid and glucocorticosteroids, and immunomodulatory agents, azathioprine, mercaptopurines and cyclosporine [2]. However, these drugs are unsatisfactory as repeated relapses and serious side effects often occur. Indeed, the drug-induced toxicity appears to be a continuous challenge. Over the past decades, though substantial progress has been made to treat this debilitating disease, effective therapies are yet available. Although the exact etiologies of colitis remain unknown, it is generally accepted that IBD is an autoimmune disease with an initial defect in sampling gut luminal antigens, or a mucosal susceptibility that leads to the activation of immune responses. In consequence, the overproduction of pro-inflammatory mediators from immune cells, such as reactive oxygen species, nitrogen metabolites and pro-inflammatory cytokines, result in mucosal damage and the sustenance of inflammatory responses [3] [4]. According to a number of studies, we acknowledged that various kinds of intestinal immune cells play important roles in the initiation of IBD; however, activated CD4+ T helper (Th) cells, explicitly Th1 and Th2, are definitely responsible for the maintenance of the inflammatory condition s of CD and UC [5]. It is established that CD is generally considered as a Th1 cytokine-mediated disorder whereas UC appears to be likely arisen from Th2-mediated responses. Nevertheless, some other new subsets of Th cells have also been suggested to induce the IBD pathologies [6]. Upon the intrinsic generation of different inflammatory mediators and their release into the bloodstream, tissue injury and/or epithelial damage are observed as the disrupted mucosal homeostasis, tissue injury and/or epithelial damage are observed as the disrupted mucosal homeostasis in IBD. Among these pro-inflammatory mediators, the pro-inflammatory cytokines, particularly tumor necrosis factor α (TNF-α), interleukin (IL)-1β, IL-6, IL-12 and IL-23 are believed to play crucial roles in the pathogenesis of IBD [7–9]. Therefore,the regulation of these cytokines has been proposed as a strategy to treat IBD [10]. In this regard, anti-inflammatory drugs, aminosalicylates and corticosteroids, and immunosuppressive agents, are considered as the first-line therapies for IBD patients [11]. As mentioned earlier, undesired side effects are often arisen from these mainstream pharmaceuticals, the recrudescence rates of IBD stay relatively high [12, 13]. Therefore, the use of complementary and alternative (CAM) approaches has been increasingly appreciated by the sufferers in the management of colitis.

In recent years, traditional Chinese medicine (TCM) has been considered as one of the most popular CAM approaches for IBD patients as the bioactivities of a variety of TCM herbs have been validated by bench-to-bedside studies. Intriguingly, the application of medicinal formulations, which are mixtures of herbs or phytocompounds that influence multiple systems in the body, has been shown with preferential effect over the individual conventional treatment protocols for IBD sufferers indicating that herbal medicine can be a promising alternative approach for the management of colitic conditions [14], and several medicinal formulas comprising Chinese herbs have been demonstrated effective against IBD [15].

According to the Chinese medical theory, the symptoms of UC are derived from the imbalance of *Qi* accompanied with dampness or cold-dampness in the spleen and intestine. BaiTouWeng decoction (BTWD) is a traditional Chinese herbal recipe for invigorating the intestine and restoring the imbalance of *Qi* that has been prescribed in TCM clinics for many years. To the traditional BTWD, we added in the following herbal medicines: HerbaPogostemonis, Herba Centellat and, indigo naturalis, and we named this modified formula Chang-An-Shuan (CAS). In detail, our formula CAS comprises: Pulsatilla chinensisRegel (root of *Pulsatilla Adans*, family: Ranunculaceae), Cortex Fraxini (bark of *Fraxinus chinensis Roxb,* family: Oleaceae), RhizomaCoptidis (rhizome of *Coptis chinensis Franch*, family: Ranunculaceae), HerbaPogostemonis [leaf and stem of *Pogostemon cablin(Blanco) Benth,* family: Lamiaceae], Herba Centellat [leaf of*Centella asiatica (L.) Urban,* family: Umbelliferae], and indigo naturalis[extractive of *Baphicacanthus cusia (Nees) Bremek*, family: Acanthaceae]. Recently, Dai et al. reported that BTWD-based Chinese medicine formula could greatly improve the clinical symptoms of patients with acute period of left hemicolon type-UC [16]. According to the TCM clinical experience, several ingredients of the CAS formulation are useful for some common gastrointestinal ailments [17], including nausea, vomiting, diarrhea and abdominal cramps. However,no scientific evidence of efficacy or mechanistic action of CAS on experimental colitis has been provided. Therefore, in this study, we aimed to assess the anti-inflammatory effect of CAS on 2, 4, 6-trinitrobenzenesulfonic acid (TNBS)-induced colitis in rats, and to elucidate the underlying mechanisms of its in vivo activities.

## Methods

### Animals

Male Sprague-Dawley rats aged 8–10 weeks old weighing 180 ± 40 g were obtained from the Centre of Medical Experimental Animals of Guangdong [Certificate of quality: SCXK (Yue) 2008–0002]. Rats were housed in standard laboratory cages at the Experimental Animal Center of Guangzhou University of Chinese Medicine [Center-Certificate of quality: SCSK (Yue) 2013–0002]. The rats were given access to standard diet and autoclaved distilled water ad libitum. The housing laboratory was set with 12-h (h) light/dark cycles, the cages were ventilated, and the environment was clean and quiet. The room temperature was maintained at 22 ± 1 °C. All handling of animals and experimental procedures were conducted consistent with the institutional guidelines of the Ethics Committee of the Experimental Animal Center of Guangzhou University of Chinese Medicine, China (TCM F1–2015029).

### Reagents

Salicylazosulfapyridine (SASP) was supplied by Sunve Pharmaceutical Co. Ltd. (China, cat#20130117). TNBS was obtained from Sigma Chemical Co. Ltd. (USA, cat#1001616585). ELISA kits for rat IL-1β, IL-10 and TNF-α were purchased from Wuhan Biological Engineering Co. Ltd. (China, cat#U20017513 and CSB-E08855r; cat#U20017514; cat#V18017512 and CSB-E11987r). RNAiso Plus kit was from TaKaRa Technologies Co. Ltd. (China, cat#D9108A). SYBR®Premix Ex Taq™ (TliRNaseH Plus) was purchased from Invitrogen Life Technologies Co. Ltd. (USA, cat#DRR820A). Reverse Transcriptase M-MLV (RNase H-) reagent was purchased from TaKaRa Technologies Co. Ltd. (China, cat#RR036). Anti-p38 antibody was obtained from Abcam Co. Ltd. (USA, cat#ab27986);Anti-nuclear factor-κB(NF-κB) p65 (D14E12) XP® Rabbit mAb was from Cell Signaling Technology (USA, cat#8242S);Anti-phospho-AKT (Ser473, D9E) XP® Rabbit mAb was purchased from Cell Signaling Technology (USA, cat#4060S).

### Instruments

Tecan Sunrise (TECAN technologyCo. Ltd. Austria); TP800 Real-time PCR machine (BIO-RAD Co. Ltd. USA); NiconFluorescent electronic microscope (Nikon Co. Ltd. Japan); BIO-RAD electrophoresis (BIO-RAD Co. Ltd. USA); 721BRO4320 MultiImager machine (BIO-RAD Co. Ltd. USA).

### Preparation of CAS extract

The CAS formula is comprised of 6 Chinese medicinal raw herbs, and their proportions in formula were shown in Table 1. Six Chinese medicinal raw herbs namely *Pulsatilla chinensis Regel, Cortex Fraxini*, *Rhizoma Coptidis*, *Herba Pogostemonis*, *Herba Centellat* and *Indigo naturalis* were obtained from Guang dong Kang Mei Pharmaceutical Co., Ltd. These six Chinese medicinal materials were authenticated by Prof. Hong-mei Tang (School of Chinese Medicine, Guangzhou University of Chinese Medicine, Guangzhou, China) according to the Chinese Pharmacopoeia (version 2010) [18]. Voucher specimens (Nos.TCM-0110-CAS01, TCM-0110-CAS02, TCM-0110-CAS03, TCM-0110-CAS04, TCM-0110-CAS05, TCM-0110-CAS06) are stored in our Laboratory of Medicinal Plant and Pharmacognosy, School of Chinese Medicine, Guangzhou University of Chinese Medicine, Guangzhou, China. Six herbs were mixed and grinded according to the proportions, and then decocted with boiling water at 100 g/L for 60 min twice. The filtrates were combined and concentrated in a vacuum at 45 °C, and further freeze-dried to yield the CAS extract.

**Table 1:** The composition of CAS

Chinese herbs	Alias	Composition
*Pulsatilla chinensis Regel*	Bai Tou Weng	12–31%
*Cortex Fraxini*	Qin Pi	12–15%
*Rhizoma Coptidis*	Huang Lian	1–5%
*Herba Pogostemonis*	Huo Xiang	7–11%
*Herba Centellat*	Ji Xue Cao	12–21%
*Indigo naturalis*	Qing Dai	3–10%

### Induction of acute colitis and drug intervention

Rats were randomly divided into control group, TNBS group, SASP group and CAS groups, starved for 12 h, and lightly anesthetized with 10% chloral hydrate by an intraperitoneal route. Then a flexible polyethylene catheter with an external diameter of 2 mm was inserted rectally into the colon of the rat. The tip was 6–8 cm proximal to the anus verge [19]. To the TNBS group (*n* = 10), SASP group and CAS groups, 2.5%TNBS dissolved in 50% ethanol solution (*w*/*v*) was instilled into the colon via a cannula (125 mg/kg) for the induction of acute colitis. To the control group (*n* = 10), the same volume of ethanol solution without TNBS was instilled. Rats were held in a head-down position for 2–3 min after the instillation for a better distribution of the agent in the entire colon. Upon the TNBS induction, drug intervention (CAS or SASP) was orally administered to rats for 10 days (d). SASP was used as a positive reference drug for colitis. The SASP group (*n* = 10) was treated with SASP at 0.5 g/kg/d whereas the control group were fed with normal saline. CAS extract was given to rats at either 5 g/kg/d (*n* = 10) or 0.5 g/kg/d (*n* = 10).

### Sample collection

Blood was collected from rats and serum was obtained after centrifugationat 3500×g for 10 min. Then, the rats were sacrificed. Colonic segments were excised, freed from adherent adipose tissue, rinsed with saline to remove fecal residue and blotted dry. The length of each colon sample was measured. The colon tissues were collected for protein and RNA extraction.

### Disease activity index (DAI)

The DAI of model rats was taken from day 3 till the last day of the experimental period. The index was assessed based on three variables, including the change of body weight, stool consistency, and bleeding, in accordance with a previously reported method [20].

### Histological examination of colonic tissue

When rats were sacrificed, distal colons were harvested, rinsed and fixed in 4% paraformaldehyde at 4 °C overnight. Colonic tissues were then processed with sequential clearing and dehydrating steps, and embedded in paraffin blocks. Samples were sectioned into 6 μm slices and subjected to standard Hematoxylin and Eosin (H&E) staining for the evaluation of colonic architecture, loss of crypts, extent of injury and mucosal damage and lymphocyte infiltration.

### Measurement of serum and colonic cytokine levels

The blood samples were centrifuged at 3500×g for 10 min and serum was obtained. The colon samples were collected by the same location and quality. Serum and colon samples were stored at −80 °C until assayed. Levels of IL-1β, TNF-α and IL-10 were measured with enzyme-linked immunosorbent assay (ELISA) kits according to the manufacturer’s instructions. All samples were assayed in duplicate.

### Western blotting analysis

Frozen proximal and distal colon specimens were homogenized in extraction buffer, which contained 50 mmol/L Tris-HCl (pH 8.0), 150 mmol/L NaCl, 1% Triton X-100, 0.02% sodium azide, 1 μg/mL aprotinin and 100 μg/mL phenylmethylsulfonyl fluoride (PMSF). The homogenates were centrifuged at 12000×g for 10 min at 4 °C, and protein concentration in the supernatant was quantified by bicinchoninic acid method. 60 μg of proteins were separated using 10% or 12% sodium dodecyl sulfate-polyacrylamide gel electrophoresis (SDS-PAGE), and the separated proteins were then transferred electrophoretically onto nitrocellulose membranes. After blocking the non-specific binding sites with 5% non-fat dry milk in Tris buffered saline (TBS) for 1 h, the membranes were then incubated with primary antibodies against p-38 (1:1000), p-AKT (1:1000), NF-κB p-65 (1:1000) or β-actin (1:500) respectively overnight at 4 °C. The β-actin loading was served as the internal control. The membranes were then washed with TBS with 0.1% Tween-20 (TBST) three times and incubated with appropriate secondary antibody for 1 h at room temperature. Detection of protein was achieved by enhanced chemiluminescence agent, and the blot was subjected to autoradiography.

### Real-time quantitative polymerase chain reaction (qPCR)

Total RNA was isolated from colon tissue using RNAiso Plus kit (TaKaRa technologies Co. Ltd) according to the manufacturer’s protocol. The primer sequences for 18S, TNF-α and IL-1β are listed in Table 2. The cycling parameters for one-step PCR were as follows: reverse transcription 95 °C for 30s, 95°Cfor 3 s, denaturation 60 °C for 34 s (38 cycles) on the BIO-RAD system. Duplicate cycle threshold (CT) values were analyzed in Microsoft Excel using the comparative CT method as described by the manufacturer. The amount of target (2^−ΔΔCT^) was obtained by normalizing it to the endogenous reference 18S.

**Table 2:** List of primer sequences for qPCR

Primer	Sequence	Product size
18S_forward	5′-GAATTCCCAGTAAGTGCGGGTCATA-3′	105 bp
18S_reverse	5′-CGAGGGCCTCACTAAACCATC-3′	
TNF-α_forward	5′-CTGAGGTCAACCTGCCCAAGT-3′	100 bp
TNF-α_reverse	5′-GAGAACGGATGAACACGCCAGT-3′	
IL-1β_forward	5′-CATCAGCACCTCTCAAGCAGA-3′	161 bp
IL-1β_reverse	5′-CATTCTCGACAAGGGGGCTC-3′	

### Statistical analysis

Data are expressed as the means ± standard error of mean (S.E.M.). *P < 0.05, vs. control group; #*P* < 0.05, vs. TNBS group. Statistical analysis was performed using the SPSS 19.0 statistical software. The parameters were evaluated by one-way analysis of variance (ANOVA) followed by LSD-t test. *P*-values less than 0.05 were considered statistically significant.

## Results

### CAS decreased DAI scores in TNBS-induced colitis in rats

**Fig. 1:**
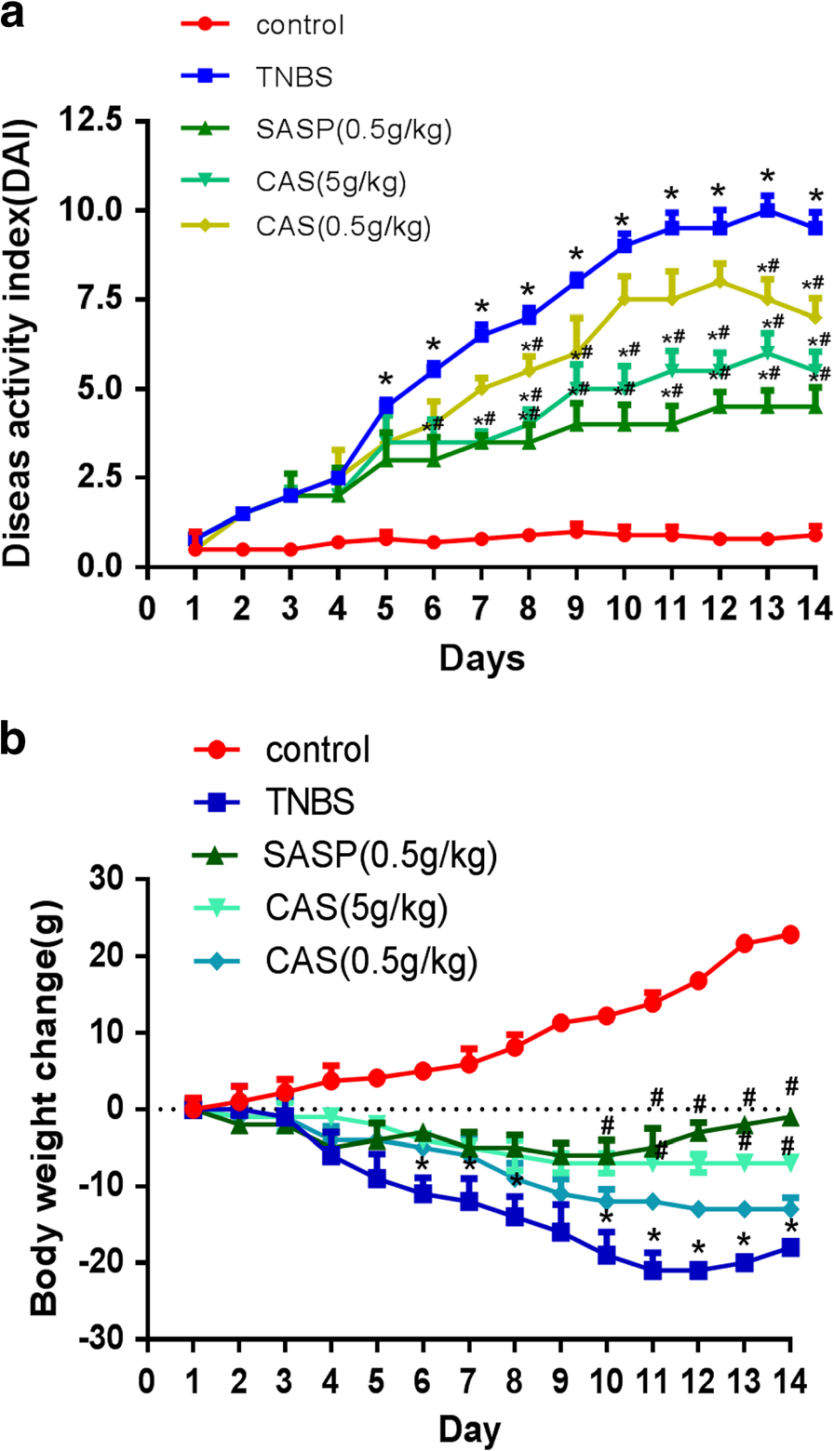
CAS decreased DAI scores in TNBS-induced colitis in rats. Compared with the control group, *P<0.05; compared with the TNBS group, #*P*< 0.05

The DAI score is a common parameter for the evaluation of the severity of colitis. The higher DAI score indicates, more severe colitis condition. Using the hemoccult sensa test as a diagnostic aid, the occurrence of bloody stool was detected in 95% of the colitis rats. Compared with the control group, the DAI score of TNBS group was significantly higher indicating that the TNBS group exhibited substantial loss of body weight, diarrhea and bloody stool. The DAI scores of the SASP group and CAS groups were repressed; however, the effect of CAS treatment at 0.5 g/kg was not considered as statistically significant (Fig. 1a). The body weight loss was remarkably improved in the SASP group and the CAS treatment group at 5 g/kg from day 6 to day 10 (Fig. 1b).

### Histological examination of colonic tissues

**Fig. 2:**
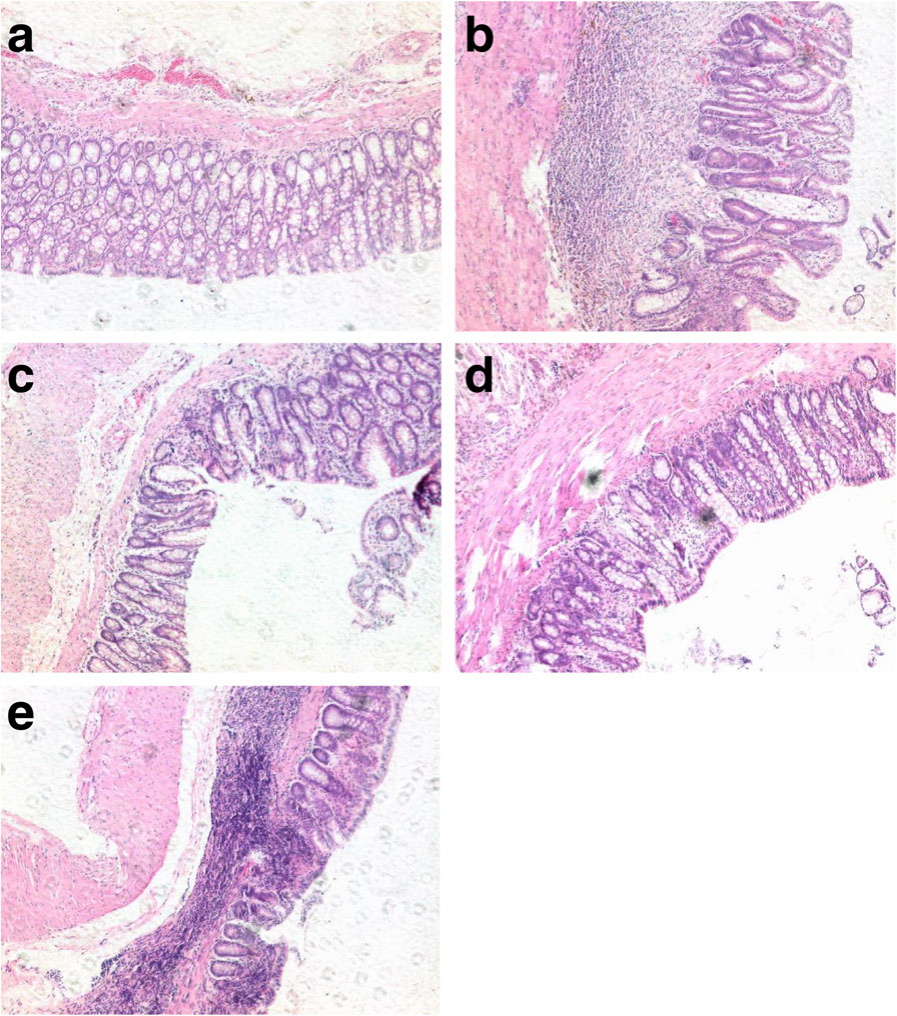
Histological examination of colonic tissues. The control group (**a**); the TNBS group (**b**), the SASP group (**c**), the CAS 5 g/kg group (**d**) and the CAS 0.5 g/kg group (**e**)

We demonstrated that colitis was successfully induced in the TNBS group as the animals were severely anorexic and subjected a marked decrease in average food intake. In addition, rats instilled with TNBS solution showed prostration, piloerection and hypomotility. From the H&E stained sections, colonic tissue of the TNBS group (Fig. 2b) exhibited disrupted colonic architecture, thickened muscle layer, inflammatory infiltrate with crypt abscesses, neutrophil-infiltrating glandular epithelia and epithelial hyperplasia when comparing to that of the control group (Fig. 2a). The SASP group (Fig. 2c) and the CAS 5 g/kg group (Fig. 2d), but not the CAS 0.5 g/kg group (Fig. 2e), showed significant improvement on mucosal architecture and normal mucosa with intact epithelial surface.

### Serum levels of IL-1β, IL-10 and TNF-α

Serum levels of pro-inflammatory cytokines, such as IL-1β and TNF-α, and anti-inflammatory cytokines, namely IL-10, were measured by [20, 21]. Means of ELISAs, as the imbalance of pro- and anti-inflammatory cytokines in the bloodstream indicates a systemic diseased condition. When compared with the control group, the levels of pro-inflammatory cytokines IL-1β and TNF-α in the serum of TNBS rats were significantly elevated by 45% and 29% respectively. Meanwhile, the serum level of anti-inflammatory cytokine IL-10 was declined by 26%. The administration of SASP and CAS at 5 g/kg notably decreased the production of systemic IL-1β and TNF-α (Table 3). Importantly, the CAS treatment significantly restored the anti-inflammatory cytokine IL-10 whereas the positive reference drug SASP exerted no significant effect (Table 3).

**Table 3:** The serum levels of cytokines in different experimental groups

Group	n	IL-1β (pg/ml)	TNF-α (pg/ml)	IL-10 (pg/ml)
Control	10	186.99 ± 28.89	113.91 ± 17.05	2.9578 ± 1.37
TNBS	10	271.33 ± 88.19*	147.34 ± 59.03*	2.2006 ± 0.56*
SASP (0.5 g/kg)	10	176.82 ± 25.42 #	112.97 ± 38.77 #	1.5980 ± 0.55 #
CAS (5 g/kg)	10	216.57 ± 20.28 #	118.63 ± 31.12 #	2.8371 ± 1.10
CAS (0.5 g/kg)	10	224.98 ± 54.92	122.19 ± 17.35	2.2244 ± 0.57

Data are reported as means ± SEM, *n* = 10. * *P* < 0.05 vs control group; # *P* < 0.05 vs TNBS group

### Colon levels of IL-1β and TNF-α

**Fig. 3:**
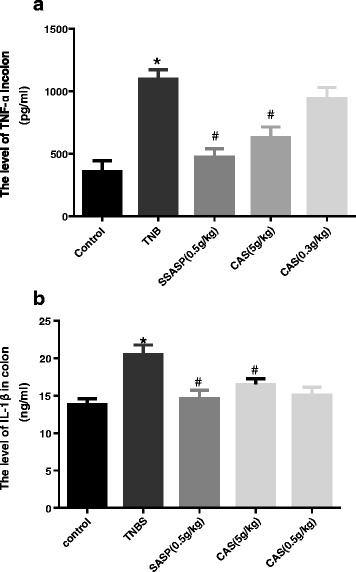
Colon levels of IL-1β (**b**) and TNF-α (**a**). Compared with the control group, *P<0.05; compared with the TNBS group, #*P*<0.05

Our ELISA kit results revealed that the levels of TNF-α (Fig. 3a) and IL-1β (Fig. 3b) in colonic samples of the TNBS group were significantly up-regulated when compared with the control group. In line with our ELISA results, the expression of TNF-α (Fig. 3a) and IL-1β (Fig. 3b) in the SASP group and the CAS (5 g/kg) group, but not the CAS (0.5 g/kg) group, were significantly suppressed.

### The expression levels of NF-κB p-65, p38 and p-AKT

**Fig. 4:**
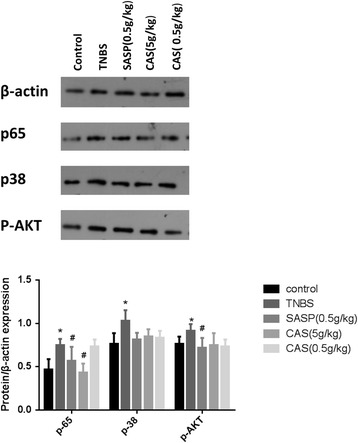
The expression levels of NF-κB p-65, p38 and p-AKT. Compared with the control group, *P<0.05; compared with the TNBS group, #*P*<0.05

Our Western blotting results demonstrated that the expression levels of NF-κB p-65, p38 and p-AKT were all up-regulated in the TNBS group when compared to the control group. Upon the intervention of SASP and CAS (5 g/kg), the protein level of NF-κB p-65 was significantly suppressed. The levels of p38 and p-AKT proteins were also reduced by SASP and CAS; however, the reductions were not statistically significant (Fig. 4).

### mRNA expression of pro-inflammatory cytokines in colonic tissues

**Fig. 5:**
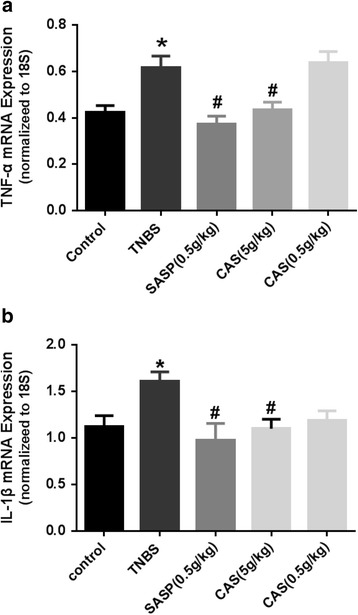
mRNA expression of pro-inflammatory cytokines in colonic tissues. Compared with the control group, *P<0.05; compared with the TNBS group, #*P*<0.05

Our real-time qPCR analysis revealed that the mRNA expression levels of TNF-α (Fig. 5a) and IL-1β (Fig. 5b) in colonic tissues of the TNBS group were significantly up-regulated by 30–35% when compared with the control group. In line with our ELISA results, the mRNA expression of TNF-α (Fig. 5a) and IL-1β (Fig. 5b) in the SASP group and the CAS (5 g/kg) group, but not the CAS (0.5 g/kg) group, were significantly suppressed.

## Discussion

Animal models of intestinal inflammation are frequently used for studying the pathogenesis of human IBD [22]. For instance, TNBS-, acetic acid- and dextran sulfate sodium-induced colitis models have been developed to help elucidate the pathogenic and molecular mechanisms of human IBD [23]. Similar to other chemically induced colitis models, TNBS is a chemical agent commonly used to induce colonic inflammation in the gut of rodents. The TNBS-induced colitis is characterized by several clinical and histopathological features resembling those of human IBD [24]. Generally, TNBS-induced colitis has been regarded a model for aninmal colitis as transmural granulomatous inflammation associated with diarrhea, rectal prolapse, weight loss and colonic wall thickening are observed [18], and is very suitable to study anti-inflammatory agents during the course of developing and resolving inflammation [19]. In the current study, CAS significantly improved the recovery of body weight loss and reduced DAI scores in TNBS-induced rats indicating that CAS, a TCM herbal formula, exerts a potent protective effect against mucosal inflammation. This study confirmed the efficacy of CAS in an experimental colitis that was associated with an inhibition of the NF-κB signaling pathway.

Although the etiology of IBD is not completely understood, it is well accepted that the imbalance between pro-inflammatory cytokines, such as TNF-α, IFN-γ, IL-1β, IL-6 and IL-12, and anti-inflammatory cytokines, namely IL-4, IL-5 and IL-10, plays a central role in the modulation of inflammatory processes [25–27]. Previous reports demonstrated that inflamed mucosa from UC patients have shown increased expression of certain pro-inflammatory cytokines such as IL-1β, IL-6 and TNF-α [26, 27]. In this study, the application of CAS significantly suppressed the production of IL-1β and TNF-α meanwhile the level of anti-inflammatory cytokine IL-10 was enhanced. To this end, the protective effect of CAS against colitis was plausibly related to its maintenance of a relative balance between pro-inflammatory and anti-inflammatory cytokines. As such, mucosal inflammation was alleviated, so as the development of IBD symptoms.

NF-κB activation is believed to be a pivotal mediator of a number of inflammatory processes as well as other vital cellular responses. The enhancement of the NF-κB subunit p65 was an indication of the activation of NF-κB [28]. In the TNBS-induced rats, NF-κB activation was significantly up-regulated [29]. Our result was in agreement with previous findings that NF-κB activation plays a critical role in most immune and inflammatory processes including the etiology of IBD [30]. Importantly, the application of CAS notably reduced the expression level of NF-κB p65. Taken together, the anti-inflammatory effect of CAS on the development of TNBS-induced colitis was related to the inhibition of the NF-κB signaling pathway. On the other hand, the PI3K-dependent AKT phosphorylation is considered as a NF-κB crosstalk signaling. Indeed, AKT is a serine/threonine kinase that is a direct downstream target of PI3K [31]. The phosphorylation of AKT generates a positive feedback in the maintenance of inflammation by activating transcriptional factors, such as NF-κB, which is a key regulator of gene transcription of various pro-inflammatory cytokines [32, 33]. The inhibition of AKT phosphorylation was therefore implicated in reducing colonic damage in IBD patients [34]. Our Western blotting result showed that AKT phosphorylation was suppressed in TNBS-treated rats, though not in a significant manner, by administration of CAS. As long as the AKT phosphorylation was not enhanced, the severity of TNBS-colitis was ameliorated. Collectively, the present study demonstrated that CAS attenuates systemic inflammatory responses via an inhibition of NF-κB activity in rats with TNBS-induced colitis. With such anti-inflammatory effect provided, we suggest that CAS is potentially a remedy for attenuating the pathological conditions in IBD.

## Conclusions

Taken together, the results reported here show that CAS is a potential alternative remedial approach for treating IBD conditions, and the anti-inflammatory effect of CAS is associated with the down-regulation of the NF-κB signaling pathway and the balanced production of pro- and anti-inflammatory cytokines.
